# Isolation and Characterization of a Deoxynivalenol-Degrading Bacterium *Bacillus licheniformis YB9* with the Capability of Modulating Intestinal Microbial Flora of Mice

**DOI:** 10.3390/toxins12030184

**Published:** 2020-03-15

**Authors:** Shiwei Wang, Qiuqiu Hou, Qianqian Guo, Jian Zhang, Yanmei Sun, Hong Wei, Lixin Shen

**Affiliations:** 1 Key Laboratory of Resources Biology and Biotechnology in Western China, Ministry of Education, College of Life Sciences, Northwest University, Xi’an 710069, China; wangsw@nwu.edu.cn (S.W.); HouQiuq@163.com (Q.H.); sheldonamy@139.com (Q.G.); zhangjian940706@163.com (J.Z.); sunyanmei2001@126.com (Y.S.); 2 State Key Laboratory of Agricultural Microbiology, College of Animal Sciences and Technology, Key Laboratory of Agricultural Animal Genetics, Breeding, and Reproduction of the Ministry of Education & Key Laboratory of Swine Genetics and Breeding of Ministry of Agriculture and Rural Affairs, Huazhong Agricultural University, Wuhan 430070, China

**Keywords:** deoxynivalenol, degradation and detoxification, *Bacillus licheniformis* YB9, intestinal microbial flora

## Abstract

Deoxynivalenol (DON) is one of the most prevalent food- and feed-associated mycotoxins. It frequently contaminates agricultural commodities and poses serious threats to human and animal health and leads to tremendous economic losses globally. Much attention has been paid to using microorganisms to detoxify DON. In this study, a *Bacillus licheniformis* strain named YB9 with a strong ability to detoxify DON was isolated and characterized from a moldy soil sample. YB9 could degrade more than 82.67% of 1 mg/L DON within 48 h at 37 °C and showed strong survival and DON degradation rate at simulated gastric fluid. The effects of YB9 on mice with DON intragastrical administration were further investigated by biochemical and histopathological examination and the gut microbiota was analyzed by 16S rRNA Illumina sequencing technology. The results showed that DON increased the levels of aspartate aminotransferase (AST), alanine aminotransferase (ALT), and creatinine (Cr), decreased those of immunoglobulin G (IgG) and IgM in serum, and resulted in severe pathological damage of the liver, kidney, and spleen. By contrast, YB9 supplementation obviously inhibited or attenuated the damages caused by DON in mice. In addition, YB9 addition repaired the DON-induced dysbiosis of intestinal flora, characterized by recovering the balance of Firmicutes and Bacteroidetes to the normal level and decreasing the abundance of the potentially harmful bacterium *Turicibacter* and the excessive *Lactobacillus* caused by DON. Taken together, DON-degrading strain YB9 might be used as potential probiotic additive for improving food and feed safety and modulating the intestinal microbial flora of humans and animals.

## 1. Introduction

Mycotoxins are the toxic secondary metabolites of fungi and often contaminate agricultural commodities and cause serious safety issues to humans and animals due to their high occurrence incidence globally [1,2]. Deoxynivalenol (DON), notorious as vomitoxin, is one of the most prevalent trichothecene mycotoxins and is frequently detected in agricultural commodities with high concentrations [3]. DON is produced mainly by some *Fusarium* species and DON in cereals can directly or indirectly pose severe health threats to both humans and animals through the food chain and lead to tremendous economic losses worldwide.

The toxicity of DON primarily depends on its epoxide group, and it can bind with ribosomal subunit to impair eukaryotic protein synthesis and consequently cause serious damage to human and animal organs [4,5]. It has been reported that DON consumption at high acute doses resulted in a series of diseases, such as emesis, nausea, diarrhea, and food refusal. Long exposure of DON causes reduced growth and causes negative effects on the liver, spleen, kidneys, intestine, gastrointestinal tract, and so on [6,7,8,9]. The hepatotoxicity, nephrotoxicity, and immunotoxicity of DON were widely investigated in recent years [10,11]. DON exposure increased the relative weight of liver and caused up-regulation of pro-inflammatory cytokines TNF-α and IL-1β as well as the occurrence of mild fibrosis and piecemeal necrosis in the liver [11,12,13]. In regard to kidneys, DON increased the creatinine (Cr) level of serum, which is one main indicator for evaluating renal function [14,15]. In addition, it was found that the immune system was sensitive to DON and DON indigestion decreased the immunoglobulin G (IgG) and IgM levels of serum, impaired cell-mediated and humoral immunity, and increased susceptibility to infectious diseases [16,17,18]. Dietary DON with high doses caused apoptosis and necrosis of T-cells, B-cells, and leukocytes, which would suppress innate immune functions [19,20]. The atrophy of spleen was also observed after DON supplementation in chicken diets [21]. The homeostasis of intestinal microflora in animals has also been reported to be affected due to DON supplementation in diets [11,22]. DON administration decreased the diversity and richness of gut microbiota and increased the abundance of *Parabacteroides* and *Enterobacter*, but reduced those of *Odoribacter* and *Lachnospiracea incertae sedis* [14].

Many methods have been developed to eliminate DON and among them, biodetoxification of DON by using microorganisms showed great potential due to low cost, convenient operation, and being environmentally-friendly [23,24,25]. For example, it was found that a population of microbes from agricultural soil completely transformed DON into de-epoxy DON after 60 h incubation. Furthermore, this culture was found to consist of at least six bacterial genera, mainly including *Enterococcus, Serratia, Streptomyces, Stenotrophomon, Clostridium, and Citrobacter* [26]. Single microbe was also used to remove DON and it has been reported that a Gram-negative bacterial strain *C. freundii* A47 encoded DON de-epoxidizing gene and showed de-epoxidation activity [27]. However, the detoxification efficiency of some microbes is limited. In addition, there were few reports about the microbial applications for DON detoxification in feed or grain [28].

In order to obtain more high-efficient DON-removal bacteria for application in feed or grain, in this study, we isolated a *Bacillus licheniformis* strain YB9 from moldy soil. It was found that YB9 was able to efficiently remove DON by degradation *in vitro* and *in vivo*. In addition, YB9 could repair damage to liver, kidney, and spleen caused by DON and modulate the DON-induced dysbiosis of the gut microbiota in mice.

## 2. Results

### 2.1. Isolation and Identification of the DON-Degrading Strain Bacillus Licheniformis YB9

**Figure 1:**
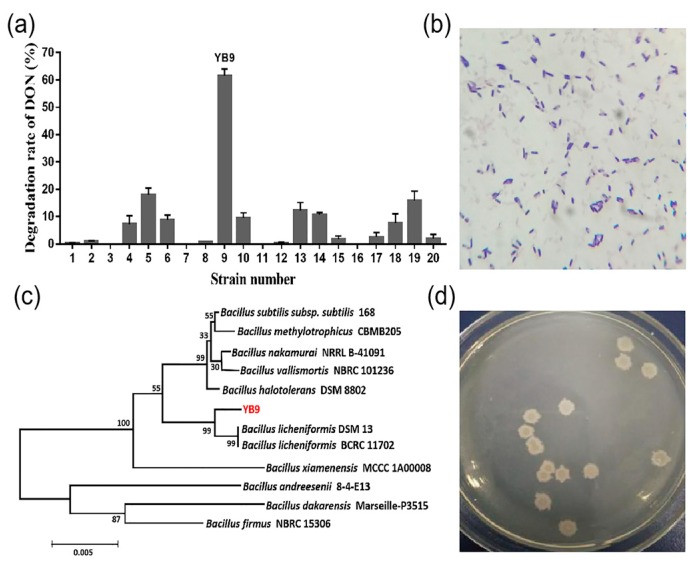
Isolation and identification of *Bacillus licheniformis* YB9. (**a**) The growth of YB9 on LB plates containing 1 mg/L DON. (**b**) Morphology and Gram-staining of YB9 observed under a microscope. (**c**) Phylogenetic tree of *B. licheniformis* YB9 based on the 16S rRNA gene sequence. (**d**) Colony morphologies of YB9 on LB.

**Figure 2:**
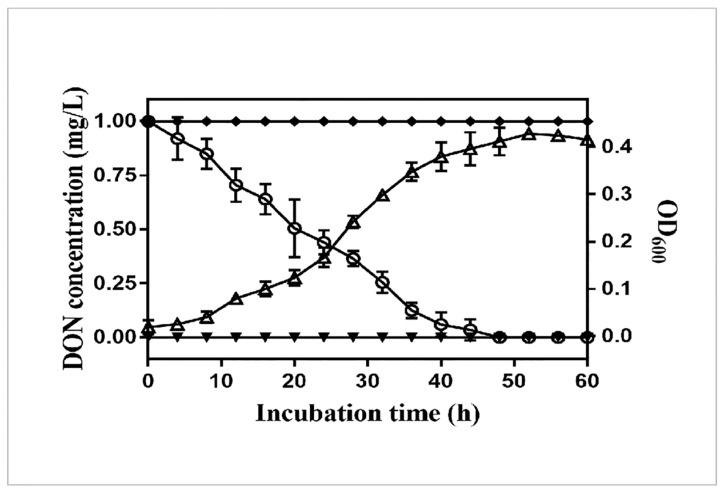
YB9 growth (empty triangles) and DON concentration (empty circles) in liquid MM containing YB9 and supplemented with DON as the sole carbon. Liquid MM containing DON (solid circles) and liquid MM containing YB9 (solid triangles) are used as controls.

More than 20 colonies that could grow on LB agar plate containing 1 mg/L DON were screened from the moldy samples. Among these bacteria, the strain named YB9 exhibited the highest removal rate of DON and removed 61.6% of 1 mg/L DON in LB broth within 48 h at 37 °C (Figure 1a). Furthermore, after cultural optimization, the removal rate of DON in LB broth increased to 82.67%. We also determined the growth and DON degradation rate of YB9 in liquid MM with 1 mg/L DON as the only carbon source by OD_600_ measurement and the DON Plus Test Kit, respectively. As shown in Figure 2, the rapid growth of YB9 was coupled with the reduction of DON levels. After 48 h, no DON could be detected and OD_600_ of YB9 showed no obvious increase. This result showed that DON might be utilized by YB9 as the sole carbon source and the removal of DON was mainly through the degradation of YB9.

Furthermore, the general biological feature of YB9 was investigated. YB9 was a Gram-positive bacterium with endospore formation, and its cells are rod-shaped under microscope (Figure 1b). It exhibited a rough colony morphology with a milky white surface and irregular edges on an LB agar plate (Figure 1d). The biochemical characteristics shown in Table 1 indicate that YB9 belonged to the genus *Bacillus*. Next, to obtain more information on the taxonomic status as well as the phylogenetic position of YB9, the construction of phylogenetic tree based on 16S rRNA gene sequence was carried out. The result revealed that YB9 had the most similarity with *B. licheniformis* DSM 13 and BCRC 11702, indicating that YB9 belonged to *Bacillus licheniformis* (Figure 1c).

### 2.2. Survival and DON Degradation of YB9 in SGF

**Figure 3:**
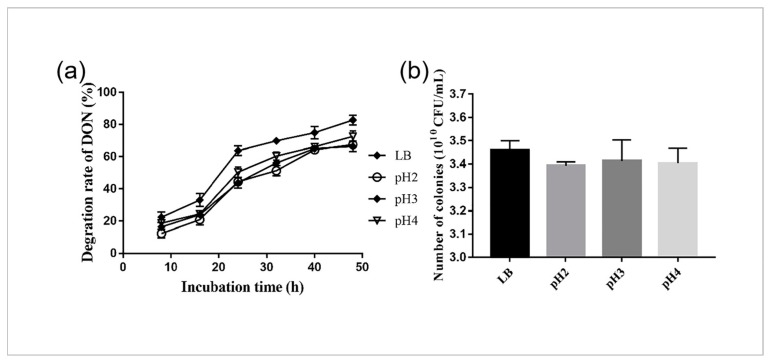
Degradation of DON and the tolerance of YB9 to SGF. (**a**) Degradation of DON by YB9 in LB and SGF during a 48 h period. (**b**) Survival of YB9 in SGF at pH 2, 3, 4, and LB (as control).

In order to determine the DON degradation of YB9 in acidic conditions similar to the digestive tracts of animals or humans, we further measured the concentration of DON in SGF at pH 2, 3, and 4. As shown in Figure 3a, the concentrations of DON were decreased in LB and SGF, and YB9, respectively, degraded 67%, 66%, and 72% of DON in SGF at pH 2, 3, and 4, and 82.67% of that in LB for 48 h. There was no significant difference in the degradation rate between SGF and LB (*P* > 0.05), and a similar result was obtained for survival rate investigation (Figure 3b). Taken together, these results indicate the application potential of YB9 on animals or humans.

### 2.3. Protection of YB9 Against the Damage of Liver, Kidney, and Spleen Caused by DON

**Figure 4:**
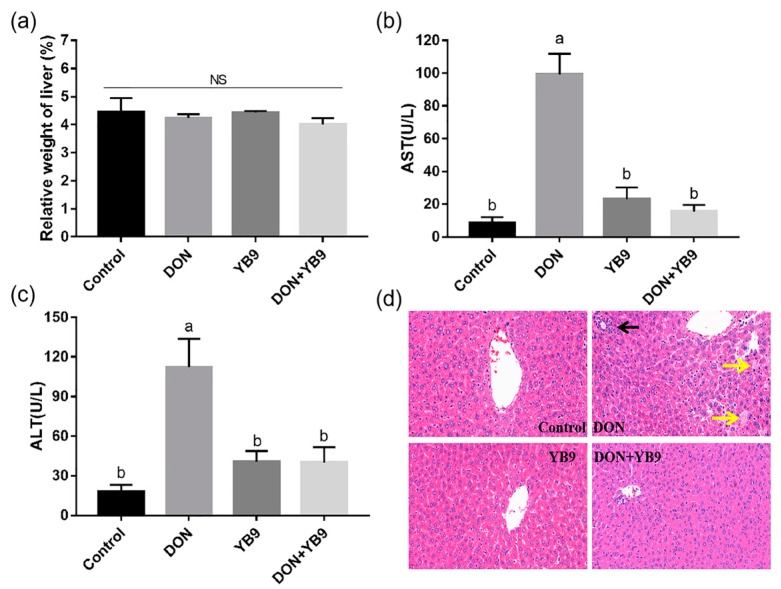
YB9 protects against DON-induced liver damage. (**a**) Relative weight of liver. (**b**) AST activity and (**c**) ALT activity of mice treated with normal saline (control group), 5 mg/kg BW DON (DON group), 7 × 10^8^ CFU/ml YB9 (YB9 group) or 5 mg/kg BW DON plus 7 × 10^8^ CFU/ml YB9 (DON+YB9 group), which were administered for 2 weeks. The different letters at the top of the columns mean significant difference (*P* < 0.05). NS, no significance. (**d**) Histopathological examination of liver tissues by HE staining. Magnification times, 400 ×.

It has been reported that DON is harmful to the liver, the main toxin-degrading organ in animals. We first investigated the effects of DON and YB9 on the liver of mice via evaluating the relative weight of liver, the AST and ALT levels of serum, and the histopathological parameters. The results showed that the relative weight of the liver in the DON group was comparable to that of the control group, while the serum AST and ALT levels increased significantly (*P* < 0.05) (Figure 4a–c). In addition, DON induced inflammatory infiltration (the black arrow) and hyaline degeneration (the yellow arrow) according to the hepatic histomorphology analysis (Figure 4d). These results indicated that DON caused the damage of the liver and, therefore, possibly impaired its function. In contrast, the relative weight of liver, the serum AST and ALT levels, and the histopathological parameters of the liver in the YB9 group were similar to those of the control group (Figure 4). In the DON+YB9 group, the supplementation of YB9 obviously recovered all the above tested indicators of the liver to the levels of the control group, such as a normal morphology of hepatocyte, and similar AST and ALT. In summary, these results indicated that YB9 could significantly inhibit the adverse effects caused by DON on the liver of mice.

**Figure 5:**
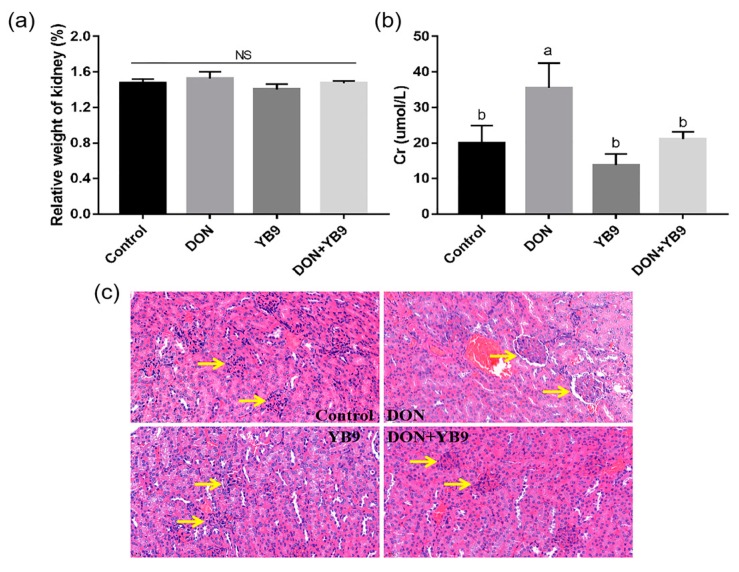
YB9 protects against DON-induced kidney lesion. (**a**) Relative weight of kidney. (**b**) Cr activity of mice treated with normal saline (control group), 5 mg/kg BW DON (DON group), 7 × 10^8^ CFU/ml YB9 (YB9 group) or 5 mg/kg BW DON plus 7x10^8^ CFU/ml YB9 (DON+YB9 group), which were administered for 2 weeks. The different letters at the top of the columns mean significant difference (*P* < 0.05). NS, no significance. (**c**) Histopathological examination of kidney tissues by HE staining. Magnification times, 400 ×.

It has been reported that after oral administration of DON in animals, high concentration of DON could be detected in kidneys, which indicated that the kidney is an organ that is targeted by DON [17]. In comparison to the control group, the relative weight of kidney has no obvious change, but the serum Cr level in the DON group significantly increased (Figure 5a,b). In addition, kidney lesion was obvious and enlargement of the renal corpuscle (the yellow arrow) was observed (Figure 5c). However, there was no significant difference between the YB9 group and the control group, indicating that YB9 had no adverse effect on kidney. Furthermore, in the DON+YB9 group, the serum Cr level was restored to the level of the control group and no obvious histological lesion of kidney was observed. The results demonstrated that YB9 administration played a positive role in protection against kidney damage due to DON addition.

**Figure 6:**
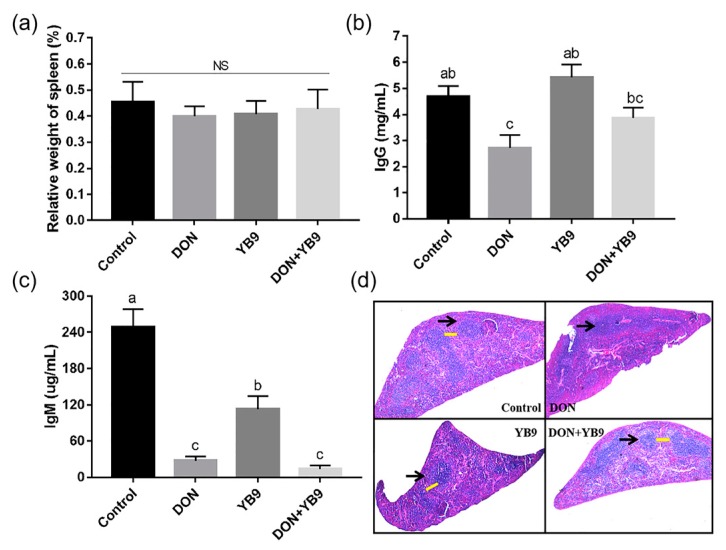
Protection of YB9 against the injury of spleen caused by DON. (**a**) Relative weight of spleen. (**b**) IgG and (**c**) IgM activity of mice treated with normal saline (control group), 5 mg/kg BW DON (DON group), 7 × 10^8^ CFU/ml YB9 (YB9 group) or 5 mg/kg BW DON plus 7x10^8^ CFU/ml YB9 (DON+YB9 group), which were administered for 2 weeks. The different letters at the top of the columns mean significant difference (*P* < 0.05). NS, no significance. (**d**) Histopathological examination of spleen tissues by HE staining. Magnification times, 40 ×.

Furthermore, we examined the effects of DON and YB9 on spleen, the largest immune organ of mice. The results showed that DON exposure decreased the serum IgG and IgM levels compared with the control group, while the relative weight of spleen in all the samples had no significant difference (*P* > 0.05) (Figure 6a–c). In addition, the atrophy of spleen, diffuse white pulp expansion (the black arrow), and the disappeared marginal zone (the yellow arrow) in spleen were observed in the DON group (Figure 6d). These results indicated that DON induced spleen injury and affected the immune function of mice. Compared with the control group, the serum IgG level of the YB9 group was similar, while the serum IgM level was slightly decreased. The histomorphology result of the spleen in the YB9 group was similar with that of the control group except for mild atrophy (Figure 6d). In regard to the DON+YB9 group, no pathological changes mentioned above in the spleen were observed by microscopic examination. Its histological result and the level of IgG but not IgM were restored to the control levels due to the YB9 addition (Figure 6b,c). The results indicated that YB9 could alleviate DON toxicosis on the spleen of mice.

### 2.4. Effects of DON and YB9 on the Intestinal Flora of Mice

Many researchers have found that intestinal flora affects the overall health of the host [14,21], therefore we investigated the influence of DON and YB9 on gut microbiota in colon tissue of mice by the analysis of 16S rRNA sequencing.

**Figure 7:**
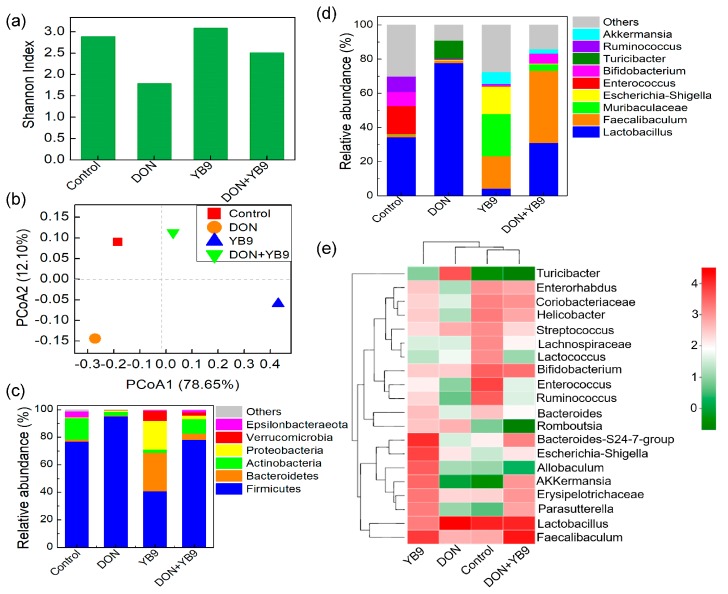
Effects of DON and YB9 on the intestinal flora of mice. (**a**) Shannon analysis based on alpha diversity of intestinal flora. (**b**) PCoA analysis based on β-diversity of intestinal flora. (**c**) The relative abundance at the phylum level. (**d**) The relative abundance at the genus level. (**e**) The heatmap analysis of the top-20 relative abundance of the genus in all the samples.

The sequencing coverage of the samples ranged from 99.3% to 99.8% and indicated that the sequencing depth was enough to reflect the structure and composition of the intestinal microbes of mice. Shannon is an index of alpha diversity. As shown in Figure 7a, the Shannon index of the DON group was the lowest among the four samples and no apparent difference was observed among the control group, YB9 group, and DON+YB9 group. Principal coordinate analysis (PCoA) indicates Beta diversity of samples and it was performed according to the composition of OTUs in the different treatment groups. The PCoA results showed that the DON+YB9 group was closer to the control group (Figure 7b).

As is well-known, *Firmicutes* and *Bacteroidetes* were the two dominant phyla in intestinal flora. Similarly, *Firmicutes* showed the highest abundance among all the samples. DON reduced the abundance of *Bacteroidetes* and increased that of *Firmicutes*. In contrast, YB9 administration increased the abundance of *Bacteroidetes* and reduced that of *Firmicutes*. In addition, the rate of *Firmicutes* to *Bacteroidetes* in the DON+YB9 group was similar to the control group (Figure 7c).

At the top-10 abundance genus level, the abundances of bacterial genera in the DON group were obviously different with that of the control group (Figure 7d) and particularly increased the abundances of *Lactobacillus* and *Turicibacter*, a potentially harmful bacterium [25]. In YB9 group, the abundance of *Lactobacillus* was decreased while that of *Faecalibaculum* was increased relative to that of the control group. However, the bacterial abundance, especially *Lactobacillus* and *Turicibacter* in the DON+YB9 group, were similar to those of the control group, although the abundance of *Faecalibaculum* increased (Figure 7d). Similar results were obtained by heatmap analysis (Figure 7e). In addition, the heatmap result showed that the bacterial composition in DON+YB9 was more similar to that of the control group. Taken together, these results show that YB9 could largely repair DON-induced disorder of intestinal flora.

## 3. Discussion

DON has received more and more attention in recent years because it causes a variety of serious safety issues and economic losses in humans and animals due to its high incidence of contamination and occurrence on crops and feeds globally [3,9]. Therefore, methods need to be urgently developed to eliminate DON for preventing these adverse effects. As is well-known, biological detoxification of DON by microorganisms or enzymes was a preferred choice due to its advantages in high specificity and effectivity [23,24]. In this study, we isolated and identified *B. licheniformis* YB9 from moldy soil, which exhibited high DON-degrading ability in vitro and in vivo. YB9 could degrade 82.67% of 1 mg/L DON at 37 °C for 48 h in LB. In addition, YB9 could grow in liquid MM using DON as a sole carbon resource, indicating that the removal of DON was mainly through degradation. Some microorganisms with the ability to degrade DON have been reported and they could transform DON into other non-toxic or less toxic compounds such as de-epoxy DON(DOM-1), 3-epi-deoxynivalenol (3-epi-DON), 3-keto-deoxynivalenol (3-keto-DON) [23,29]. The detailed mechanisms of DON degradation by YB9 is worthy to investigate in the future. YB9 belongs to *Bacillus* and can produce spores with high resistance to environmental stress, such as high heat, ultra violet light, and low pH solution. Here, YB9 was obtained after heat treatment at 80 °C for 15 min. In addition, many *B. licheniformis* have been proved to be safe as probiotics in food and feed [30,31]. Taken together, these results indicated an application potential of YB9 during food or feed process and storage.

It has been reported that DON contamination in the diet could bring about severe damage to the liver, spleen, and kidney of humans or animals [10,11]. DON-degrading strains are expected as an additive in crops or feeds to improve quality and safety. Therefore, we investigated the DON degradation effects of YB9 on mice by examining the relative weight of organs and biochemical and histopathological parameters after intragastrical administration. Consistent with previous results, DON could induce different degrees of damage of the liver, kidney, and spleen in mice [9,16,32], as was indicated by some biochemical indices of serum and histological examination. In contrast, YB9 had no obvious effects on the liver, kidney, and spleen of mice and most of the indicators were similar with the control group, except the reduced IgM level. In the DON+YB9 group, all the changed parameters caused by DON, including the serum levels of AST, ALT, Cr, and IgG in mice, were restored to the levels of the control group except IgM. These results suggested that YB9 might have slightly negative effects on the spleen and the reasons for this need further research. In short, the damages from DON administration were significantly repaired after adding YB9 into the DON group.

DON not only causes organ damage, but also alters the intestinal flora of animals, which is closely associated with many diseases, such as metabolic diseases, immune system diseases, and other diseases [11,14]. The effects of YB9 and DON on the intestinal flora of mice were investigated in this study. DON exposure reduced the diversity and notably altered the composition of gut microbiota in mice, which increased the abundance of *Firmicutes* and decreased that of *Bacteroidete* compared with the control group. The results are consistent with previous studies [22] and the high ratio of *Firmicutes* to *Bacteroidetes* might be harmful to human health [33]. In contrast, YB9 had no significant effects on gut microbiota and furthermore, in DON+YB9 group supplementation of YB9, restored the diversity of gut microbiota and the ratio of *Firmicutes* to *Bacteroidetes* in mice. At the genus level, DON obviously increased the relative abundance of *Lactobacillus* and *Turicibacter* from the phyla *Firmicutes.* It has been reported that the high relative abundance of *Turicibacter* was observed in the patients or mice with inflammatory bowel disease [25], indicating that *Turicibacter* might be a potential harmful bacterium. *Lactobacillus* is recognized as a potential beneficial bacterium for its health-promoting effects on gut microbiota [33], but more than a 2-fold abundance of *Lactobacillus* in the DON group was observed compared with the control group. Excessive *Lactobacillus* has adverse effects on the intestinal tract, such as intestinal discomfort of the host [34]. As observed in the DON+YB9 treatment, YB9 supplementation could restore the balance of *Firmicutes* and *Bacteroidetes* to the normal levels of the control. In addition, YB9 addition increased the abundance of *Faecalibaculum* and decreased the potential harmful bacterium *Turicibacter* and the excessive *Lactobacillus* due to DON supplementation. In summary, YB9 could largely renormalize and maintain the homeostasis of intestinal flora destroyed by DON, but the detailed mechanisms need to be further investigated.

In summary, our findings showed that the DON-removal strain *B. licheniformis* YB9 could be used as a potential probiotic additive for improving food and feed safety and positively regulating the homeostasis of intestinal flora, which provides theoretical basis for practical application in the future.

## 4. Materials and Methods

### 4.1. Culture Media and Reagents

For DON-removing bacteria isolation, Luria-Bertani (LB) agar plus 1 mg/L DON was used. For growth and DON degradation rate analyses, minimal medium (MM) plus DON as the only carbon source was used, which comprised 1 g/L NH_4_NO_3_, 1 g/L NaCl, 1.5 g/L Na_2_HPO_4_, 0.2 g/L MgSO_4_·7H_2_O, 1.0 g/L KH_2_PO_4_, and 1 mg/L DON. Unless otherwise indicated, all the reagents that were used in this study were analytical grade and purchased from Sigma Chemical Co. (Sigma-Aldrich, St. Louis, MO, USA). The biochemical assay kits for serum index examination, such as the activity of aspartate aminotransferase (AST) and alanine aminotransferase (ALT), Cr, IgG, and IgM, were purchased from Nanjing Jiancheng Bioengineering Institute (NJJCBIO, Nanjing, China). The DON Plus Test Kit obtained from Romer Labs Inc (AgraQuant, Newark, DE, USA) was used.

### 4.2. Isolation of Microorganisms with DON-Removal Ability

A moldy soil sample from the experimental farm of Army Medical University (Chongqing, China) was collected and 10 g of soil was added to 100 mL of phosphate saline buffer (PBS) and mixed by vortex. The mixture was then heated in a water bath of 80 °C for 15 min. The supernatant was diluted appropriately with PBS and the dilutions were immediately spread on LB plates containing 1 mg/L DON. The plates were incubated at 37 °C for 24 h. The colonies obtained were individually transferred to LB broth containing 1 mg/L DON and cultured at 37 °C in a shaking bath at 200 rpm for 48 h. The residual levels of DON were determined using the DON Plus Test Kit according to the manufacturer’s instructions (Romer Labs, Newark, DE, USA). In brief, 100 μL of the samples or standards were added to each color-coded dilution well with 200 μL of conjugate and mixed. Then, 100 μL of mixture was transferred to antibody-coated wells and incubated for 15 min at room temperature. After being washed with PBST (PBS containing 0.5% Tween) for five times and dried on absorbent paper towel, 100 μL of substrate solution including 3,3’,5,5’-tetramethylbenzidine was added to each well and incubated for 5 min at room temperature. Subsequently, 100 μL of stop solution was added to each well. The optical density values at 450 nm (OD_450_) were detected on a plate reader and then the levels of DON were calculated according to the standard curve. Bacterial isolates with DON-removal ability were preserved in 20% sterile glycerol and stored at −80 °C.

In order to further determine if the potential strains with DON-removal ability were tolerant or able to degrade DON, 200 μL of overnight culture was transferred into 10 mL of liquid MM containing 1 mg/L DON as the only carbon source. Liquid MM only containing DON or the strain were used as controls. DON concentration together with the growth of the strain were respectively measured by DON Plus Test Kit and the optical density at 600 nm (OD_600_) every 4 h for 60 h. Each treatment contained three replicates and the data were displayed as the average values of three replicates.

### 4.3. Characterization of the DON-Degrading Strain YB9

The morphological observation of the strain named YB9 with high DON-removal ability was carried out using a microscope on an LB agar plate. Physiological and biochemical characteristics of the bacterium were routinely determined by Gram-staining as well as physiological and biochemical tests according to the Common Bacterial System Identification Manual [35]. To identify YB9 at the molecular level, PCR-based 16S rRNA amplification was performed using the universal primers 27F (5′-AGAGTTTGACCTGGCTCAGTTACGACT-3′) and 1492R (5′- GGTTACCTTGTTACGACTT-3′). The purified PCR product was then sequenced at BGI Inc. (Shenzhen, China). The nucleotide sequence including the hypervariable V3 to V4 regions was analyzed by a BLAST search against the NCBI database. A phylogenetic tree was then constructed using MEGA 5.0 program [36].

### 4.4. DON Degradation and Survival of YB9 in Simulated Gastric Fluid (SGF)

DON degradation of YB9 under gastric acid condition was determined using SGF, which consisted of 3.2 g/L pepsase and 2 g/L NaCl. SGF was adjusted using HCl until the values of pH were 2, 3, or 4. YB9 was inoculated into LB broth and cultivated with shaking at 37 °C for 12 h. Subsequently, 200 μL of YB9 culture was added to 10 mL of SGF solution or 10 mL of LB with 1 mg/L DON. The equal volume of SGF or LB broth without YB9 was used as negative controls. All the samples were cultivated at 37 °C until 48 h. The concentration of DON was respectively measured by the DON Plus Test Kit every 8 h.

In order to determine the survival of YB9 under gastric acid condition, the growth of YB9 was investigated after exposure to SGF with pH 2, 3, and 4. YB9 was inoculated into LB broth and cultivated with shaking at 37 °C for 12 h. Then, the mixture with 200 μL of YB9 and 10 mL of SGF or 10 mL of LB (as control) was incubated at 37 °C for 4 h. The culture was sampled and serially diluted with PBS. Subsequently, 200 μL of the dilutions were transferred to an LB agar plate to examine YB9 growth. Each sample had three replicates.

### 4.5. Design of Animal Experiments

All animal experimental procedures were approved by the Laboratory Animal Care Committee of the P. R. China Ministry of Health and complied with ethical standards (Certificate NO. ACCNU-2017-0024, 15 April 2017).

Three-week-old Balb/C mice were obtained from the Animal Experimental Center of Army Medical University (Chongqing, China) and were used to investigate the effects of DON and YB9 on liver, kidney, spleen, and intestinal flora of colon tissue. All the mice were housed in sterile Trexler-type plastic film isolators under a controlled environment (21 to 22 °C, 45–55% relative humidity, and every 12 h light-dark cycle) and were permitted free access to sterilized water and feed as previously described [35]. The water and bottles were high-pressure-steam sterilized at 121 °C for 30 min, and the bedding and food were sterilized by 40-kGy Co-60 gamma irradiation. After 7 days acclimation, 40 mice were individually weighed and then randomly and equally allocated to four treatments. According to previous animal modes of DON [28] and our pre-experiments, the mice were intragastrically administered with the following treatments once a day for 2 weeks: control group, 400 μL of normal saline; DON group, 200 μL of DON (5 mg/kg BW) + 200 μL of normal saline; YB9 group, 200 μL of YB9 culture with 7 × 10^8^ CFU/mL + 200 μL of normal saline; DON+YB9 group, 200 μL of DON (5 mg/kg BW) + 200 μL of YB9 culture with 7 × 10^8^ CFU/mL. All the mice were subjected to fasting overnight during the end period of the experiments.

#### 4.5.1. Organ Weight

All the mice were individually weighed (body weight) and then sacrificed. Their liver, kidney, and spleen were dissected and weighted, respectively. All organ weights were expressed relative to body weight (g/g × 100%).

#### 4.5.2. Biochemical Parameter Examination

After being sacrificed, the blood samples of the mice were collected in 1.5 mL Eppendorf tubes containing 5 U heparin by eyeball enucleation. After being kept for 1 h at room temperature, approximately 150 μL of serum was separated from whole blood by centrifugation at 1500× *g* for 10 min and stored in tubes at −80 °C for further analysis. The biochemical parameters of serum, AST, ALT, Cr, IgG, and IgM were respectively determined by biochemical assay kits from Nanjing Jiancheng Bioengineering Institute (NJJCBIO, Nanjing, China) following the manufacturer’s instructions. Taking the detection of ALT, for example, firstly 20 μL of alanine transarninase matrix solution and 5 μL of serum sample were added to each well and plated in water bath at 37 °C for 30 min. Then, 20 μL of 2,4-dinitro-phenylhydrazine solution was added and incubated for 20 min in water bath at 37 °C. Subsequently, 200 μL of NaOH solution was added. After 15 min at room temperature, the OD_510_ values of wells were measured by a plate reader. The serum levels of AST, ALT, Cr, IgG, and IgM were respectively calculated according to the corresponding standard curve.

#### 4.5.3. Histopathological Examination

The obtained tissues were respectively dissected, fixed in paraformaldehyde, and then embedded in paraffin. The embedded tissues of each mouse were sliced into 4-μm-thick pieces and then subjected to haematoxylin and eosin (HE) staining for histological examination. A section of each tissue from each animal was evaluated by a certified veterinary pathologist who was blinded to the treatments under microscope. For each sample, 3 to 5 areas at 4 different sections were analyzed.

#### 4.5.4. Determination of the Effects of YB9 and DON on Gut Microbiota

The colon tissues were sterilely collected from the intestinal tract, immediately frozen in liquid nitrogen, and then stored at −80°C. The contents of the colon from each treatment were sent to Majorbio (Shanghai, China) for 16S rRNA Illumina sequencing and the analyses were performed as previously described [36].

### 4.6. Statistical Analysis

The experimental data were analyzed using the Statistical Analysis System software (SAS) package version 9.1 as previously described [37]. In brief, all the results are expressed as the means ± standard deviations. The analysis of organ relative weights, AST, ALT, Cr, IgG, and IgM levels in mice was conducted with a 2 × 2 factorial design using PROC GLM in SAS 9.1. All statements of significance were based on probabilities of *P* < 0.05 and *P* < 0.01.

## Figures and Tables

**Table 1:** Biochemical characteristics of YB9.

Substrate/Test	Result	Substrate/Test	Result
Sugar fermentation	+	Amylohydrolysis	+
3%H_2_O_2_	+	7% NaCl	+
Semi-solid agar	+	Indole	−
Nitrate	+	Citrate utilization	+
Methyl red test	−	pH 5.5–8.7	+
V-P	+	Casein hydrolysis	+
Yolk	−		

Note: “+” indicates that the test result is positive, “−” indicates that the test result is negative.
